# Water quality measurements in San Francisco Bay by the U.S. Geological Survey, 1969–2015

**DOI:** 10.1038/sdata.2017.98

**Published:** 2017-08-08

**Authors:** Tara S. Schraga, James E. Cloern

**Affiliations:** 1 U.S. Geological Survey, 345 Middlefield Rd., Menlo Park, California 94025, USA

**Keywords:** Marine chemistry, Ecosystem ecology, Environmental monitoring

## Abstract

The U.S. Geological Survey (USGS) maintains a place-based research program in San Francisco Bay (USA) that began in 1969 and continues, providing one of the longest records of water-quality measurements in a North American estuary. Constituents include salinity, temperature, light extinction coefficient, and concentrations of chlorophyll-*a*, dissolved oxygen, suspended particulate matter, nitrate, nitrite, ammonium, silicate, and phosphate. We describe the sampling program, analytical methods, structure of the data record, and how to access all measurements made from 1969 through 2015. We provide a summary of how these data have been used by USGS and other researchers to deepen understanding of how estuaries are structured and function differently from the river and ocean ecosystems they bridge.

## Background & Summary

On April 10 and 11, 1969 oceanographers from the U.S. Geological Survey (USGS) conducted the first hydrographic research cruise along the salinity gradient of San Francisco Bay (SFB)—one of the largest estuaries on the west coast of the Americas. Although it was not the researchers’ original intention, that survey launched an observational program that continues and expanded into a program of long-term ecosystem research that has contributed to the development of estuarine oceanography as a scientific discipline. In that era little was known about how estuaries function as transitional ecosystems between land and sea, where seawater and fresh water meet. Early USGS studies focused on: estuarine circulation where surface waters flow seaward over a landward-flowing bottom layer^1^; sediment accumulation in an estuarine turbidity maximum^2^; geomorphology^3^; marsh vegetation and land forms^4^; biogeochemistry of nutrients, oxygen and carbon^5^; benthic invertebrate communities^6^; urban pollution^7^, and its flushing by river inflows^8^.

Over time the research expanded into new domains to measure, model and understand: tidal circulation and transport processes^9–11^; human modifications of sediment supply^12^ and geomorphology^13^; sediment-water nutrient exchanges^14,15^; microbial biogeochemistry^16,17^; bioaccumulation and cycling of contaminants including petroleum hydrocarbons^18^, metals^19^, mercury^20^, PCBs^21^, and selenium^22–24^; disturbance by introduced species^25,26^; ecosystem metabolism^27^; phytoplankton communities^28^, productivity^29^, and regulating processes^30,31^; zooplankton ecology^32^; responses to climate variability^33,34^ and climate change^35^.

**Figure 1:**
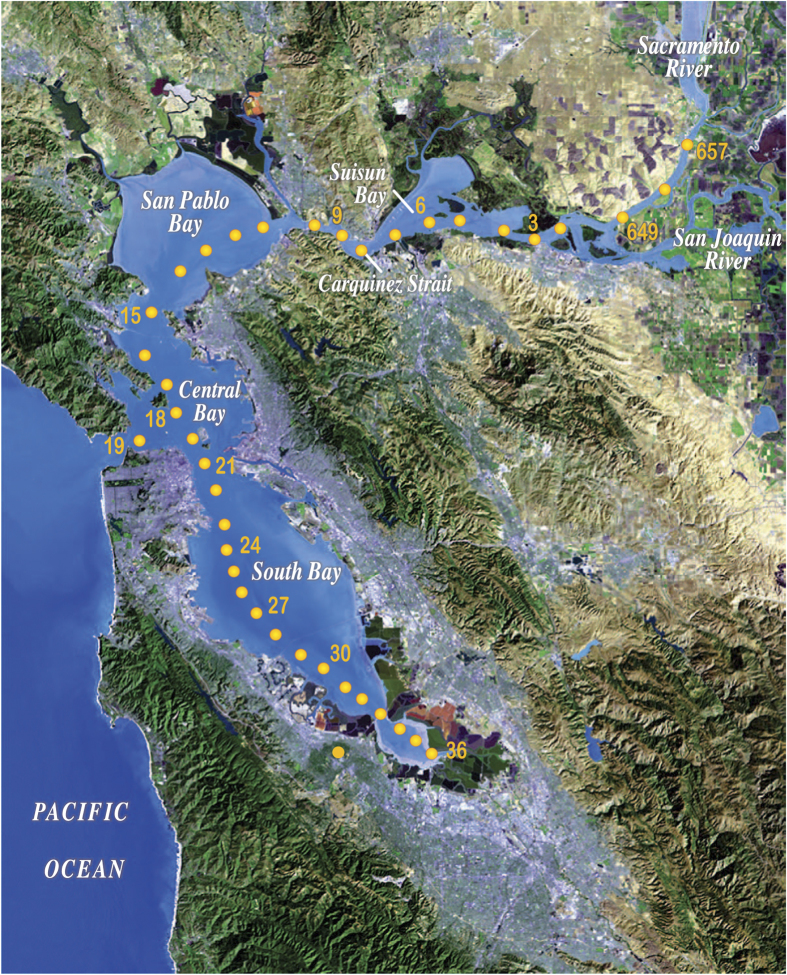
Map showing locations of USGS sampling stations in San Francisco Bay. Station coordinates are given in Table 1.

Central to this research is a core set of measurements repeated over time at a network of sampling sites (Fig. 1, Table 1) spaced along the estuarine salinity gradient. The San Francisco Bay system has been a useful place for studying estuarine dynamics because it includes two different estuary types. South Bay (stations 21–36) is an urbanized marine lagoon, and North Bay (stations 15–657) is the estuary of California’s two largest rivers, the Sacramento and San Joaquin. Central Bay connects South and North Bays to each other and to the coastal Pacific Ocean (Fig. 1). Thus, one goal of USGS research has been to compare two different estuary types^36^. The data set includes measurements of salinity, temperature, suspended particulate matter, light penetration, dissolved oxygen, chlorophyll-*a* as an indicator of phytoplankton biomass, and concentrations of dissolved inorganic N, P and Si. The sampling program maps longitudinal and vertical distributions of these estuarine properties and captures their variability at seasonal, annual and decadal time scales.

The data described here were collected for one research purpose—to measure and understand how an estuarine ecosystem changes in response to human activities and the climate system. However, we recognized from the beginning of this effort that the data have value beyond this one purpose. We have encouraged and supported use of these data by others, and the diversity of applications of this data set has been both surprising and gratifying. We illustrate this diversity with examples of scientific articles (Table 2 (available online only)) that used the data for purposes we could not have imagined, ranging across disciplines of archaeology, geochemistry, hydrodynamics, ecotoxicology, conservation biology, sediment dynamics, and biology of organisms from microbes to seabirds. Some of these publications were collaborations with visiting scientists, postdocs and graduate students. Others were done independently of USGS research. The collective knowledge accumulated from this research over decades has contributed to the global understanding of estuaries as ecosystems situated where land, ocean, atmosphere and people converge. Our purpose here is to widen accessibility of these data so their value continues to grow.

## Methods

USGS water-quality studies in San Francisco Bay include two types of measurements: (1) laboratory analyses of discrete water samples collected aboard ship (chlorophyll-*a*, dissolved oxygen, suspended particulate matter, dissolved inorganic nutrients), and (2) shipboard or submersible sensors to measure salinity, temperature, chlorophyll fluorescence, dissolved oxygen, turbidity, and light attenuation. The analyses of discrete water samples were used to calibrate the chlorophyll fluorescence, dissolved oxygen, and turbidity sensors, with individual calibrations for each sampling cruise, and often separate calibrations for each bay region. Therefore, the data record includes both discrete measurements (e.g., Discrete_Chlorophyll-*a*) and sensor-based in-situ measurements (e.g., Calculated_Chlorophyll-*a*).

From 1969 through March 1987 the discrete water samples were collected by submersible pump that delivered bay water to a shipboard fluorometer, nephelometer, thermistor, and conductivity sensor^37^. Vertical profiles were obtained by lowering the pump to prescribed depths, typically 0, 2, 5, 10, 20 m. Since April 1987 the discrete water samples have been collected near surface (~1.5 m) by pump and ~1 m above bottom with a Niskin bottle, and vertical profiles of salinity and temperature have been obtained with a Sea-Bird Electronics SBE-9 CTD. In 1988 turbidity, chlorophyll-*a* fluorescence, and photosynthetically active radiation (PAR) sensors were added to the CTD package, and in 1993 a dissolved oxygen sensor was added. In 2002 we started using a Sea-Bird Electronics SBE-9plus CTD. The individual sensors on the CTDs changed over time as new technologies emerged (see below). The CTD system is lowered through the water column at a rate<1 m s^−1^, collecting >24 samples/meter for Seabird sensors and >5 samples/meter for third party sensors. The values we report are averages over 1-m depth bins centered at the depth reported (i.e., CTD values are means of all measurements made 0.5 m above and 0.5 m below the reported depth).

### Sampling design

**Figure 2:**
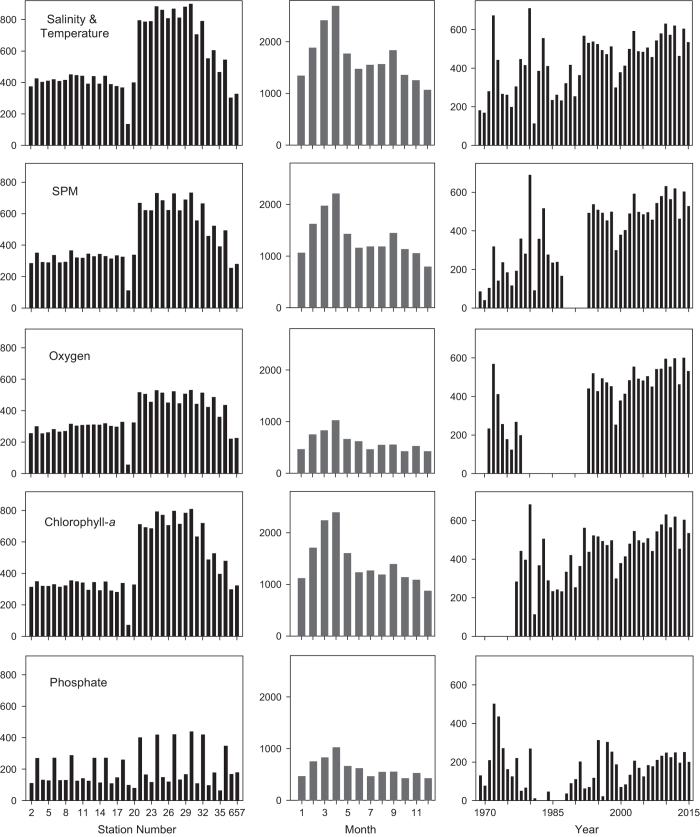
Sampling effort to measure variability of salinity and temperature, suspended particulate matter, dissolved oxygen, Chlorophyll-*a*, and dissolved inorganic nutrients (e.g., phosphate) in San Francisco Bay from 1969–2015. Plots show the number of samples (y-axis) collected at each station, in each month, and in each year.

This data set was acquired as a component of a research program whose goals evolved over time, so the frequency, spatial coverage, and makeup of water-quality measurements varied from year to year. We characterize sampling effort for five constituents, measured as the number of samples binned by station, month, and year (Fig. 2). Sampling effort was greatest in South Bay and during March and April, reflecting a key research objective to follow dynamics and ecological and biogeochemical consequences of the spring phytoplankton bloom^30^. The record reveals multi-year gaps in measurements of SPM and dissolved oxygen; that chlorophyll-*a* measurements first began in 1977; and that nutrient (e.g., phosphate) concentrations were measured less frequently than other constituents (Fig. 2). Sampling became more regular, and all constituents were measured each cruise starting in 1993 when this program became incorporated into the Regional Monitoring Program for Water Quality in San Francisco Bay (http://www.sfei.org/rmp).

### Depth

Pump depth was determined using a pressure transducer with an accuracy of +/− 1 m. Readings at zero meters are representative of a 0.4 m intake depth. Beginning March 1987, depth was determined with a Paroscientific Digiquartz (http://www.paroscientific.com/) pressure transducer as part of a Sea-Bird Electronics CTD package.

### Discrete_Chlorophyll-*a*

Chlorophyll-*a* measurements began in 1977, and methods changed as new instrumentation and widely-accepted standard methods emerged. Samples were collected onto Gelman GFF (glass fiber) filters and pigments were extracted with 90% acetone. The absorbance of the extracts was measured with a Varian 635D spectrophotometer following Strickland and Parsons^38^. Chlorophyll-*a* concentrations were calculated using the SCOR-UNESCO trichromatic equations^39^. Beginning in 1983, we used Lorenzen’s^40^ spectrophotometric equations. In 1992 we began using a Hewlett Packard 8452A diode array spectrophotometer. In 1999 we began measuring chlorophyll-*a* concentrations fluorometrically using the acidification method on a Turner Designs TD-700 fluorometer calibrated with chlorophyll-*a* standard^41,42^. Since 2011 we have used a Turner Designs Trilogy fluorometer. After each method change we compared results of the older and newer approach on replicate samples across a range of chlorophyll-*a* concentrations to verify that bias was not introduced as new instruments and methods were used.

### Calculated_Chlorophyll-*a*

Vertical profiles of chlorophyll-*a* were derived from calculated concentrations based on calibrations of an in-vivo fluorometer done each cruise to account for variability of phytoplankton species assemblages and the relationship between chlorophyll-*a* and fluorescence^43^. Calibrations were linear regressions of Discrete_Chlorophyll-*a* (above) and in-vivo fluorescence measured initially with a Turner Designs Model 10 fluorometer connected to a pumped stream of bay water. Beginning in 1988, profiles were obtained with a SeaTech fluorometer connected to a Sea-Bird Electronics CTD. This was replaced with a Turner Designs SCUFA fluorometer in 2002, and with a Turner Designs C7 fluorometer in May 2004.

### Discrete_DO

Water samples for dissolved oxygen measurement were collected into 300-ml BOD bottles that were filled from the bottom and allowed to overflow at least 3 times their volume. Winkler reagents^38^ were added immediately and bottles were stored capped with water in their cap-wells. In the laboratory, 100.2 ml of acidified sample was titrated manually following Carpenter^44^. Beginning in 1993, the samples were analyzed with a Metrohm 686 titroprocessor autotitrator^38^ using the potentiometric titration method of Granéli and Granéli^45^. Potassium iodate standardization of the sodium thiosulfate was conducted (Knapp *et al.*, 1991). In 2007 the autotitrator was replaced with a Metrohm Titrino 798.

### Calculated_DO

In 1993 we added a Sea-Bird Electronics SBE-13 sensor to the CTD package to obtain vertical profiles of dissolved oxygen. The sensor was calibrated prior to each cruise with 100% and zero saturation endpoints, and additionally with Discrete_DO measurements (above) each cruise. In 2002 we began using a Sea-Bird Electronics SBE-43 oxygen sensor calibrated each cruise with Discrete_DO measurements.

### Discrete_SPM

Suspended particulate matter was measured gravimetrically as mass collected onto pre-weighed 0.45 μm pore-size silver filters (1969–1984) or polycarbonate 0.4 μm pore-size membrane filters (1993–2015). A correction was made for mass of salt retained on the filter^46^.

### Calculated_SPM

Vertical profiles of suspended particulate matter were calculated concentrations derived from individual calibrations of a nephelometer or optical backscatter sensor each cruise. Calibrations were linear regressions of Discrete_SPM (above) and voltage output from a Turner Designs Model 10 fluorometer configured as a nephelometer connected to the pumped stream of bay water^37^. Beginning 1993, SPM profiles were obtained with a D&A Instrument Company (now Campbell Scientific) OBS-3 optical backscatter sensor as part of a Sea-Bird Electronics CTD package.

### Extinction_coefficient

PAR (mol quanta m^−2^ s^−1^) was measured using a Li-Cor Biosciences LI-192 underwater quantum sensor (1977–1982, 1988–2015). The light extinction coefficient (*k*) was computed from the slope of the regression of ln(PAR) against water depth. Measurements were initially made at 6–7 depths per station. From 1983 through 1987 the light extinction coefficient was computed from Secchi depth SD (m) using an empirical relationship derived for San Francisco Bay: *k*=0.4+109/SD^47^. Beginning in 1988, the LI-192 sensor was deployed as part of a Sea-Bird Electronics CTD package collecting at least 28 measurements per meter, generating high-resolution vertical profiles of PAR.

### Salinity

Salinity was initially measured with an Industrial Instruments RS5-3 induction salinometer (accuracy of 0.3 PSU), and beginning December 1969 with a CM^2^ model 516 CTD probe^48^. Output from that probe was verified each cruise with 6–12 duplicate water samples analyzed in the laboratory with a Beckman RS7-B salinometer calibrated with Copenhagen water (agreement of 0.2 PSU)^48^. Beginning July 1974, salinity was measured with an electrodeless induction salinometer, with outputs validated each cruise with duplicate samples run on the Beckman salinometer (agreement of 0.05 PSU)^48^. In March 1987 we began measuring salinity with a Sea-Bird Electronics SBE-4 conductivity sensor as part of the CTD package, and since 2002 with a SBE-4C conductivity sensor. Salinity was computed from conductivity, temperature, and pressure^48^.

### Temperature

Temperature was initially measured with linear thermistors calibrated at ice point and near 20 °C each cruise^49^. Beginning March 1987, temperature was measured with a Sea-Bird Electronics SBE-3 temperature sensor as part of the CTD package, and since 2002 with a SBE-3plus temperature sensor.

### Nutrients

Samples for dissolved inorganic nutrient analyses were filtered through polycarbonate 0.4-μm pore size membrane filters into bottles previously acid washed with 10% HCL. From 1971–2003, most sample bottles were pre-washed with acetone and 2.5 meq l^−1^ sodium bicarbonate instead of 10% HCL, with blank analyses confirming undetectable nutrient concentration. Sample filtrates were either analyzed immediately, refrigerated and analyzed within 48 h, or frozen until analysis. Frozen samples were allowed to thaw at room temperature for at least 14 hours before analysis. Samples were analyzed with a Technicon II AutoAnalyzer for: dissolved silica using Technicon Industrial Method 105-71WB^50^, dissolved reactive phosphate using the method of Atlas *et al.*^51^ with ascorbic acid as a reductant, nitrate+nitrite using Technicon Industrial Method AII 100-70 W^52^, and ammonium using the method of Solorzano^53^ and starting in 1980 with color development at 37 °C following Berg and Abdullah^54^. Standards for each analyte were prepared in artificial river water and artificial seawater^38^. Beginning in April 1991, the ammonium method was modified to improve precision as detailed in Hager^46^. Beginning in 2006, nutrients were stored frozen until analysis by the Richard Dugdale laboratory at San Francisco State University using a Bran and Luebbe AutoAnalyzer II for all nutrients except ammonium, which was analyzed by spectrophotometer. Nitrate, nitrite, and phosphate were determined according to Whitledge *et al.*^55^. Silicate was determined with Bran Luebbe AutoAnalyzer Method No. G-177-96 (ref. 56). Ammonium was determined with the method of Solorzano^53^. Beginning in March 2014, nutrients were analyzed by the USGS National Water Quality Laboratory with a Thermo Scientific Aquakem 600 automated discrete analyzer using methods of Fishman and Friedman^57^ for nitrite, phosphate, and silicate, the method of Patton and Kryskalla^58^ for nitrate, and the Solorzano method^53^ for ammonium with a salt correction factor applied^59^.

## Data Records

The dataset includes the following fields for each record:

Date: format MM/DD/YY

Station_Number: locations shown in Fig. 1 and provided in Table 1

Depth: sampled depth below the surface (m)

Discrete_Chlorophyll-*a*: chlorophyll-*a* measured in a water sample (μg l^−1^)

Calculated_Chlorophyll-*a:* chlorophyll-*a* calculated from in-vivo fluorescence (μg l^−1^)

Discrete_Oxygen: dissolved oxygen measured in a water sample (mg l^−1^)

Calculated_Oxygen: dissolved oxygen calculated from an oxygen sensor (mg l^−1^)

Discrete_SPM: suspended particulate matter measured in a water sample (mg l^−1^)

Calculated_SPM: suspended particulate matter calculated from a turbidity sensor (mg l^−1^)

Extinction_Coefficient: light extinction coefficient (m^−1^)

Salinity: Practical Salinity Units (PSU)

Temperature: water temperature (°C)

Nitrite: nitrite concentration (μM)

Nitrate+Nitrite: sum of nitrate and nitrite concentration (μM)

Ammonium: ammonium concentration (μM)

Phosphate: phosphate concentration (μM)

Silicate: silicate concentration (μM)

### Data record 1

The dataset includes 210,826 records, each representing a water sample from a unique date, station, and depth. All measurements made between 4/10/69 and 12/16/15 are available in one csv file (SanFranciscoBayWaterQualityData1969-2015v3.csv) uploaded to the USGS ScienceBase repository (Data Citation 1). An xml-formatted metadata file is also available at that repository.

## Technical Validation

Results from each sampling cruise were examined carefully by at least two members of the research team to ensure that all values fell within expected ranges, to verify that calibration regressions were an acceptable basis for computing quantities from shipboard sensor measurements, to ensure completeness of each cruise data report, and to verify that values transcribed from field notes were accurate. The complete 1969–2015 data set was validated with three steps: (1) range tests to ensure that the measured values fell within ranges that are plausible and consistent with knowledge of San Francisco Bay and other estuaries; (2) pattern tests of time series of all measurements to ensure they followed plausible and understandable patterns of variability over time; (3) pattern tests of all measurements by sampling station to ensure they followed plausible and understandable spatial patterns.

**Figure 3:**
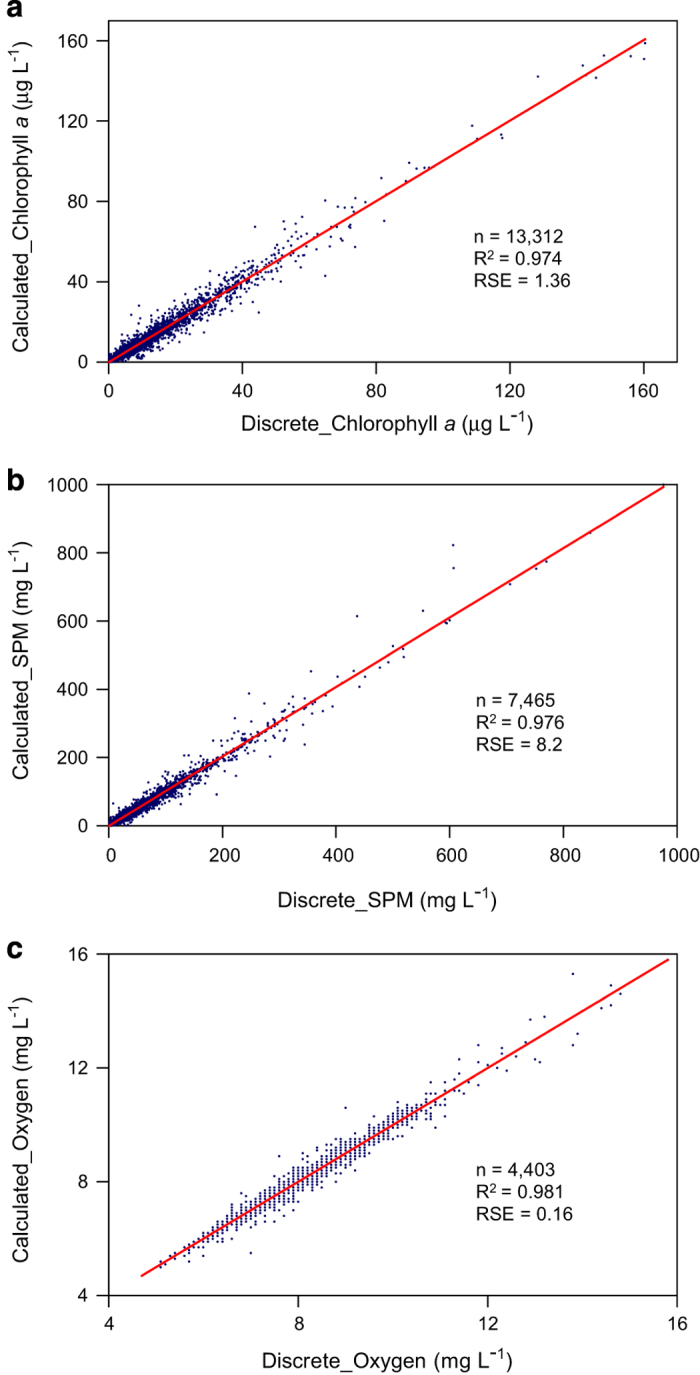
Linear relationships between all paired discrete and calculated measurements of (a) Chlorophyll-*a,* (b) suspended particulate matter, and (c) dissolved oxygen concentrations. Discrete measurements are laboratory analyses of water samples; calculated values are derived from ship-based sensors calibrated each sampling cruise with discrete measurements. Red lines are linear regressions; n, number of paired samples; R^2^, adjusted correlation coefficient from linear regression; RSE is the residual standard error of the regression, an estimator of error in calculated concentrations from sensors.

Sea-Bird Electronics sensors were calibrated annually by the manufacturer and have initial accuracies of: temperature=±0.001 °C, conductivity=±0.0003 mS m^−1^, pressure=±0.015% of full range, dissolved oxygen=±2% of saturation (http://www.seabird.com). Li-Cor LI192 sensors were calibrated by the manufacturer and sensitivity is typically 4 μA per 1,000 μmol m^−2^ s^−1^ (https://www.licor.com). Cruise-specific calibrations of shipboard fluorometers, nephelometer/optical backscatter, and oxygen sensors yielded highly significant (*P*<10^−16^) linear relationships between all discrete and calculated concentrations of chlorophyll-*a*, SPM and DO (Fig. 3). Median absolute deviations between discrete and calculated concentrations were: 0.40 μg l^−1^ for chlorophyll-*a*; 2.10 mg l^−1^ for SPM; 0.10 mg l^−1^ for DO. Linear regressions yielded residual standard errors between discrete and calculated concentrations of: 1.36 μg l^−1^ for chlorophyll-*a*; 8.2 mg l^−1^ for SPM; 0.16 mg l^−1^ for DO (Fig. 3).

Discrete chlorophyll-*a* values are mean concentrations in replicate (2, 3, or 4) aliquots from each sample. If the replicate results differed by more than 10% of their mean the results were not included in the data set. The mean coefficient of variation between replicate aliquots from 3,564 chlorophyll-*a* samples collected between 2005 and 2013 was 2.4%. Agreement between all replicates was within the recommended guideline for the method: >90% of the coefficients of variation (CV) between samples are <5% (ref. 42). Discrete suspended particulate matter precision was 1%-10%. Analytical precision of the potentiometric DO method is <0.3% (ref. 45).

**Figure 4:**
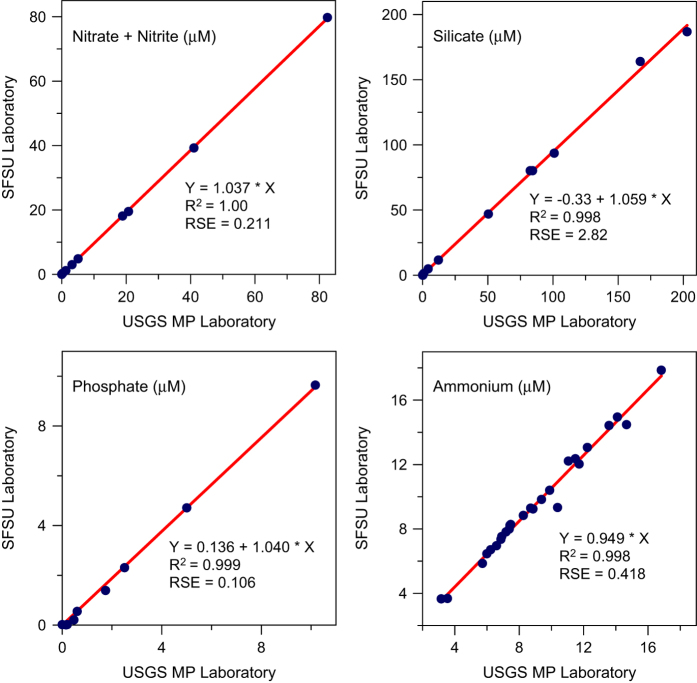
A 2005 comparison of nutrient concentrations measured in dilution series of standards (Nitrate+Nitrite, Silicate, Phosphate) or in water samples collected in San Francisco Bay (Ammonium) by two laboratories. USGS Menlo Park (USGS-MP) and San Francisco State University (SFSU). Linear regressions include an intercept if it was statistically significant. Adjusted R^2^ and Residual Standard Errors (RSE) are shown for each regression.

Nutrients analysed at the USGS Menlo Park (USGS-MP) laboratory from 1969–2005 had a typical precision of 0.02–0.2 μM for ammonium, 0.04–0.17 μM for nitrate+nitrite, 0.01–0.05 μM for nitrite, 0.01–0.05 μM for phosphate, and 0.06–1.0 μM for silicate^46,60–63^. Beginning in 2006, nutrients were analyzed by the Richard Dugdale laboratory at San Francisco State University (SFSU). That transition began after verification of acceptable agreement in analyses of nutrient standards or San Francisco Bay samples by the USGS-MP and SFSU laboratories (Fig. 4). Ammonium was analysed using different methodologies, but replicate samples analysed in each laboratory confirmed consistency between them (Fig. 4). The SFSU laboratory reported detection limits as 0.05 μM for ammonium, nitrite, nitrate+nitrite and phosphate, and 0.1 μM for silicate.

**Figure 5:**
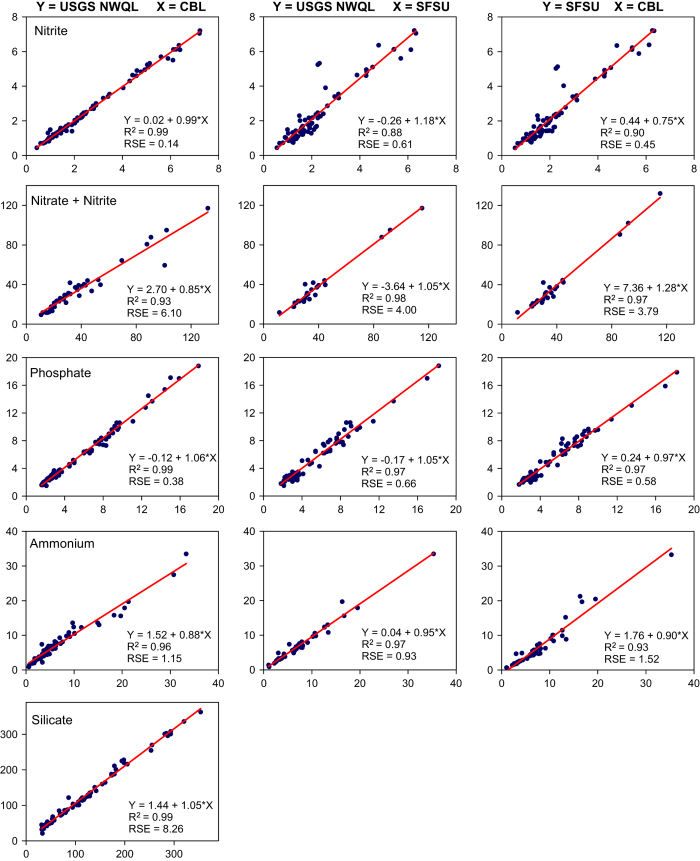
A comparison of nutrient concentrations in San Francisco Bay water samples collected from 2013–2015 and measured by three laboratories. USGS National Water Quality Laboratory (USGS NWQL), San Francisco State University (SFSU), and Chesapeake Biological Laboratory (CBL). Each comparison includes the linear regression equation, adjusted R^2^, and Residual Standard Error (RSE) as an estimator of the mean difference between laboratories. Comparisons of silicate analyses were only done for USGS NWQL and CBL.

As a preliminary step in the 2014 transition from SFSU to the USGS National Water Quality Laboratory (USGS-NWQL), we collected triplicate water samples along the salinity gradient of San Francisco Bay to compare analyses by SFSU, USGS-NWQL, and the Chesapeake Biological Laboratory (CBL) as an independent laboratory. We continued analysis of duplicate samples by USGS-NWQL and CBL through 2015. We compare results of the three laboratories in Fig. 5. The USGS-NWQL has the following minimum reporting levels: 0.7 μM ammonium, 0.1 μM nitrite and phosphate, 0.7 μM nitrate+nitrite when total<10 μM, 2.9 μM when total >10 μM, and 1.0 μM silicate. Replicate samples are intermittently analysed by USGS-NWQL to measure precision. Replicates have mean coefficients of variation<5% for all nutrients: nitrite=3.1%, nitrate+nitrite=2.3%, ammonium=4.6%, phosphate=1.6%, and silicate=0.01%.

Although nutrient methods changed over time, routine analyses of blanks and standards confirmed that methods changes did not reduce analytical precision or accuracy.

## Usage Notes

This Data Descriptor identifies a csv file that contains the complete record of USGS water-quality measurements made in San Francisco Bay from 1969–2015. Users may prefer to access the data from our project web page that includes a database from which queries can be made to select and download subsets of the full data record (https://sfbay.wr.usgs.gov/access/wqdata/index.html ). This web page also provides visual displays of water-quality spatial variability for each sampling cruise, and more detail about the research project and team members.

## Additional Information

**How to cite this article:** Schraga, T. S. & Cloern, J. E. Water quality measurements in San Francisco Bay by the U.S. Geological Survey, 1969–2015. *Sci. Data* 4:170098 doi: 10.1038/sdata.2017.98 (2017).

**Publisher’s note:** Springer Nature remains neutral with regard to jurisdictional claims in published maps and institutional affiliations.

## Supplementary Material



## Figures and Tables

**Table 1:** Names, geographic locations, and depths (mean low water) at sampling stations of the USGS research program in San Francisco Bay.

**Station number**	**Name location**	**North latitude**	**West longitude**	**Water depth (m)**
657	Rio Vista	38° 9.1'	−121° 41.3'	10.1
649	Sacramento River	3.6'	48.0'	10.1
2	Chain Island	3.8'	51.1'	11.3
3	Pittsburg	3.1'	52.8'	11.3
4	Simmons Point	2.9'	56.1'	11.6
5	Middle Ground	3.6'	58.8'	9.8
6	Roe Island	3.9'	−122° 2.1'	10.1
7	Avon Pier	2.9'	5.8'	11.6
8	Martinez	1.8'	9.1'	14.3
9	Benicia	3.4'	11.1'	34.4
10	Crockett	3.6'	12.5'	17.7
11	Mare Island	3.6'	16.0'	15.5
12	Pinole Shoal	3.1'	18.7'	8.8
13	N. of Pinole Point	1.7'	22.2'	9.8
14	‘Echo’ Buoy	0.4'	24.3'	13.1
15	Point San Pablo	37° 58.4'	26.2'	22.9
16	‘Charlie’ Buoy	55.0'	26.8'	12.8
17	Raccoon Strait	52.7'	25.3'	32
18	Point Blunt	50.8'	25.3'	43
19	Golden Gate	49.1'	28.3'	91
20	Blossom Rock	49.2'	23.6'	18.2
21	Bay Bridge	47.3'	21.5'	17.4
22	Potrero Point	45.9'	21.5'	18
23	Hunter's Point	43.7'	20.2'	20.1
24	Candlestick Point	41.9'	20.3'	11
25	Oyster Point	40.2'	19.5'	8.8
26	San Bruno Shoal	38.2'	18.8'	9.8
27	San Francisco Airport	37.1'	17.5'	13
28	N. of San Mateo Bridge	36.1'	16.2'	16.2
29	S. of San Mateo Bridge	34.8'	14.7'	14.6
29.5	Steinberger Slough	34.1'	13.1'	14.6
30	Redwood Creek	33.3'	11.4'	12.8
31	Coyote Hills	31.7'	9.5'	13.7
32	Ravenswood Point	31.1'	8.0'	12.8
33	Dumbarton Bridge	30.5'	7.3'	11.6
34	Newark Slough	29.7'	5.6'	7.9
35	Mowry Slough	28.8'	4.7'	8.5
36	Calaveras Point	28.3'	4.0'	7.9

**Table 2:** Examples of published scientific investigations that used USGS measurements of water quality and hydrography in San Francisco Bay.

**Discipline**	**Measurements Used**	**Discovery/application**
Archaeology	Salinity, Temperature	Oxygen-isotopes in clam shells were used to reconstruct human landscape use during the Late Pre-historic period^64^.
Biogeochemistry	Salinity, Temperature, Chlorophyll-a, SPM, DO, Nutrients	Concentrations of dissolved Mn, Co, Zn, and Pb all increased after a phytoplankton bloom decayed^65^.
Biogeochemistry	Salinity, Temperature, Nutrients	Showed how ecosystem metabolism of San Francisco Bay varies seasonally^66^.
Biogeochemistry	Salinity, Temperature, Chlorophyll-a, SPM, DO, Nutrients	Concentrations of dissolved methyl-mercury decreased as a phytoplankton bloom developed, then increased as the bloom decayed^67^.
Biogeochemistry	Temperature, Nitrite, Nitrate	Nitrite concentration increases during summer because ammonium and nitrite oxidation become decoupled at high environmental temperatures^68^
Bivalve ecology	Salinity	The non-native clam *Potamocorbula amurensis* can complete its life cycle along most of the estuarine salinity gradient^69^.
Bivalve ecology	Salinity, Temperature, Chlorophyll-a	Seasonal reproduction of the clam *Potamocorbula amurensis* tracks seasonal patterns of the phytoplankton food supply^70^.
Conservation biology	Salinity	Salinity data were used to assess environmental controls on and strategies for conserving sea bird populations^71^.
Ecosystem Ecology	Salinity, Chlorophyll-a, DO	Data were used in a synthesis to describe spatial and seasonal patterns of estuarine variability^72^.
Ecotoxicology	Chlorophyll-a	Data were used in a model to demonstrate how phytoplankton variability affects selenium bioaccumulation by mussels^73^.
Ecotoxicology	Chlorophyll-a	Bioavailability of Cd and Zn increased during the spring phytoplankton bloom^74^.
Ecotoxicology	Salinity, SPM, Chlorophyll-a	Data used to calibrate and validate a model of selenium transport and accumulation in estuarine biota^75^
Fish Ecology	Chlorophyll-a	Discovered a population decline of northern anchovy following introduction of the non-native clam *Corbula amurensis* and loss of the summer phytoplankton bloom^76^.
Fish Ecology	Temperature	A bioenergetics model was used to calculate consumption by striped bass, an introduced species that preys on native fishes^77^.
Geochemistry	Chlorophyll-a	Showed that seasonal patterns of organic C and N in sediments track seasonal patterns of phytoplankton biomass^78^.
Geochemistry	Temperature	Used data as input to a model of copper cycling and transport^79^.
Geochemistry	Salinity, Temperature, Chlorophyll-a, Nitrate+Nitrite	Used data to measure and understand dissolved iron and iron-binding ligand distributions along the salinity gradient^80^.
Geochemistry	Salinity, Temperature, Chlorophyll-a	Used oxygen isotope ratios of phosphate to infer local sources of wastewater P along the salinity gradient^81^.
Geochemistry	Temperature	Used data as inputs to a box model for assessing long-term fate of PCBs in the Bay^82^.
Geochemistry	Salinity, DO, SPM, Nitrate+Nitrite	Deduced a wastewater source of rare-earth elements based on their co-variation with nutrient concentrations^83^.
Hydrodynamics	Salinity	Data used to initialize and validate a 3D hydrodynamic and salinity model^84^.
Hydrodynamics	Salinity	Data used to initialize a 3D hydrodynamic model to project salinity intrusion under scenarios of sea level rise^85^.
Hydrodynamics	Salinity	Data used to validate a model of salt dispersion between the coastal ocean and Bay^86^.
Hydrodynamics	Salinity	Data used to build an empirical relationship between the salinity gradient and freshwater inflow embedded in a sediment-transport model^87^.
Hydrodynamics	Salinity	Data used to initialize, calibrate and validate a 3D tidal hydrodynamic model^88^.
Hydrodynamics	Salinity	Data were used to initialize and validate a 3D tidal hydrodynamic and salinity model^89^.
Hydrodynamics	Salinity	Discovered how salt intrusion into the estuary is related to fresh water inflow^90^.
Meiofauna Ecology	Salinity, Temperature, Chlorophyll-a	Showed that abundances of benthic foraminifera increase during phytoplankton blooms^91^.
Microbial Ecology	Salinity	Ammonia-oxidizing bacteria and archaea have different abundances and spatial patterns along the salinity gradient^92^.
Microbial Ecology	Salinity, Temperature, Chlorophyll-a, SPM	Showed that bacterial metabolism co-varies with river flow and organic-matter input^93^.
Microbial Ecology	Salinity, Temperature, Chlorophyll-a, SPM, Nutrients	Measured and identified controls on nitrification rates^94^.
Microzooplankton Ecology	Salinity, Temperature, Chlorophyll-a, Nutrients	Measured anomalously low microzooplankton grazing rates in low-salinity regions of the estuary^95^.
Paleoecology	Salinity	Discovered that benthic foraminifera assemblages remained stable over a 125-ky period, but changed after a recent species introduction^96^.
Phytoplankton Ecology	Chlorophyll-a, Nutrients	Data used to assess effects of wastewater effluent on phytoplankton communities^97^.
Phytoplankton Ecology	Salinity, Chlorophyll-a, SPM, Silicate, Light Extinction	Showed that diatom primary production and Si uptake rates decreased after introduction of the clam *Corbula amurensis*^98^.
Remote Sensing	SPM	Followed sediment deposition in restored marshes using satellite reflectance data calibrated with measured sediment concentrations^99^.
Sampling Design	Salinity	The optimum sampling design for salinity monitoring spaces stations 7.5 km apart^100^
Sclerochronology	Chlorophyll-a	Used the synchrony between chlorophyll-a and d^13^C of *Crassostria gigas* shells to infer timing of invasion by this non-native oyster^101^.
Sclerochronology	Chlorophyll-a	Used d^13^C of *Crassostria gigas* shells as a proxy for phytoplankton primary productivity and bloom timing^102^.
Sediment Dynamics	Salinity	Data used to set initial conditions of a 3D hydrodynamic, wind wave, and sediment transport model^103^.
Sediment Dynamics	Salinity	Measured settling velocities of flocculated cohesive sediments along the salinity gradient^104^.
Sediment Dynamics	Salinity	Data used to infer sediment transport pathways in the coupled Bay-ocean system^105^.
Sediment Dynamics	Salinity	Discovered that the sediment supply required to restore salt marshes varies with the slope of the estuarine salinity gradient^106^.
Sediment Dynamics	SPM	Showed that suspended sediment concentrations decreased and estuarine waters cleared suddenly after 1998 (ref. 107).
Species Introductions	Salinity	Data used to assess the potential for different non-native fish species to survive if introduced to the Bay^108^.
Teaching Estuarine Hydrology	Salinity, Chlorophyll-a, SPM	Online data were used to teach a graduate-level course, *Hydrology of San Francisco Bay and Delta*^109^.
Zooplankton Ecology	Chlorophyll-a	Chlorophyll-a was used as an index of the variable food supply to zooplankton^110^.
Zooplankton Ecology	Chlorophyll-a	Copepods *Acartia* spp. selected motile ciliates and flagellates as prey, and they were most selective when food was abundant^111^.
Zooplankton ecology	Chlorophyll-a	Discovered that micro- and nanoplankton are uniformly distributed over water depth, even during periods of stratification^112^.
Zooplankton Ecology	Nutrients	Microzooplankton consumption ranged from 15% (summer) to 73% (spring) of phytoplankton biomass in low-salinity habitats^113^.
Zooplankton Ecology	Salinity, Temperature	Data used to compute temperature-regulated predation by the non-native copepod *Tortanus dextrilobatus*^114^.
Zooplankton Ecology	Salinity, Temperature, Chlorophyll-a	Data used to explore patterns and identify environmental controls on zooplankton community variability^115^.
